# Adoptive TIL Transfer in the Adjuvant Setting for Melanoma: Long-Term Patient Survival

**DOI:** 10.1155/2014/186212

**Published:** 2014-01-08

**Authors:** Amir Khammari, Anne-Chantal Knol, Jean-Michel Nguyen, Céline Bossard, Marc-Guillaume Denis, Marie-Christine Pandolfino, Gaëlle Quéreux, Sylvain Bercegeay, Brigitte Dréno

**Affiliations:** ^1^Skin Cancer Unit, CHU Hôtel-Dieu, 1 Place Alexis Ricordeau, 44093 Nantes, France; ^2^CRCNA, INSERM U892, CNRS 6299, 9 Quai Moncousu, 44093 Nantes, France; ^3^PIMESP-SEB, Hôpital St Jacques, 85 rue St Jacques, 44093 Nantes, France; ^4^Service d'Anatomie et Cytologie Pathologiques, CHU Hôtel-Dieu, 30 boulevard Jean Monnet, 44093 Nantes, France; ^5^Plateforme de Génétique des Cancers, CHU Hôtel-Dieu, 1 Place Alexis Ricordeau, 44000 Nantes, France; ^6^Unité de Thérapie Cellulaire et Génique (UTCG), CHU Hôtel-Dieu, 9 Quai Moncousu, 44093 Nantes, France

## Abstract

Two first analyses of our clinical trial on TIL as adjuvant therapy for melanoma were published in 2002 and 2007. We present here an update of the clinical results after a 17-year median followup. In this trial, disease-free patients were randomly assigned to receive either TIL/IL-2 or IL-2. The relapse-free survival (RFS) was the primary objective. Eighty-eight patients were enrolled. A new analysis performed in May 2013 did not show significant changes in RFS or OS duration. However, our first finding on the association between the number of invaded lymph nodes and TIL effectiveness was strengthened. The Cox model adjusted on this interaction showed for the first time a significant treatment effect when considering the overall population, both on the RFS and OS. Patients treated with TIL had a longer RFS (*P* = 0.023) or OS (*P* = 0.020). This study being with a very long followup (17 years), confirmed the association between TIL effectiveness and the number of invaded lymph nodes, indicating that a low tumor burden could be a crucial factor enhancing the curative effect of TIL in possible microscopic residual disease. Moreover, we confirmed that a prolonged survival was associated with the presence of specific TIL and a decrease in Foxp3 expression.

## 1. Introduction 

Therapeutic strategies for melanoma targeting the immune system gave rise to a high medical interest over the last decade. Since the first use of interferon and interleukin-2 (IL-2), two unspecific modulating agents, the intensive emphasis on immunological approaches has recently been awarded by approval of the first immunotherapeutic agent, the human monoclonal anti-CTLA-4 (Ipilimumab) antibody, for advanced metastatic stage disease by the FDA and European health agency [1].

In the field of immunotherapy, one of the main melanoma treatments is based on the adoptive transfer of T cells, relying on the use of either specific T cells (*in vitro* simply selected and amplified or engineered by transduction of high-affinity T-cell receptors or chimeric antigen receptors) or tumor-infiltrating lymphocytes (TIL). The main rationale for this approach is that melanomas are frequently infiltrated by cytotoxic and cytokine-producing CD8^+^ T cells recognizing autologous tumor-associated antigens (TAA) [2].

Rosenberg's group first described the adoptive cell transfer-based immunotherapy in humans in 1988 [3] using bulk cultures of lymphocytes derived from autologous melanoma tumors, infused together with high doses of IL-2 in metastatic melanoma patients. After this first trial, TIL transfers have been developed and improved for the selection of TIL, and the immune followup of patients treated in numerous centers worldwide demonstrated clinical response rates of about 50% or more [4]. The main advantage of TIL is the polyclonal nature of T cells which infiltrate tumor sites, leading to the recognition of multiple known and unknown tumor antigens.

Although the clinical benefit of adoptive T-cell transfer is now well established, therapeutic responses remain short and are not observed in all patients. One plausible reason is that these therapies are mainly investigated at the metastatic stage of the disease. Indeed, at the advanced stage of melanoma, it is well known that T cells administered to the patient encounter diverse and complex resistance mechanisms linked to the tumor microenvironment and cells. The worst is the melanoma status and the more the tumor burden increases, the more the immunosuppressive reaction counteracts the eradication of tumor cells by effector T cells of the immune system, which become exhausted.

The immune inhibitory mechanism could be related to the tumor itself with the expression of PD-L1/B7-H1 and indoleamine 2,3-dioxygenase (IDO), the release of cytokines such as TGF-*β* and IL-10, loss of MHC class I or class II expression [5]. Another escape mechanism is represented by regulatory T cells and the expression by TILs of inhibitory molecules such as PD-1 or CTLA-4 [6, 7]. Based on this knowledge, we postulated that the immunosuppressive state was weaker or negligible in the early stage of the disease so that the efficiency of adoptive TIL transfer could be improved.

In the field of melanoma, only a few studies on the adoptive TIL transfer were conducted at the early stage of the disease associated with a favourable immunological status and a less important immunosuppressive tumor microenvironment in contrast to the metastatic stage.

Our group focused since 1994 on the development of an adoptive therapy based on TIL in an adjuvant setting for melanoma. We assumed that an adjuvant treatment with TIL combined with s.c. IL-2 could be effective in AJCC stage III (palpable regional lymph nodes) melanoma patients who did not yet have shown clinical evidence of metastases. A randomized open trial was conducted to assess the effectiveness of TIL + IL-2 in patients with regional melanoma lymph node metastases, but without any detectable visceral metastases. The primary endpoint of the study was to verify the effect of TIL + IL-2 treatment on the relapse-free survival (RFS) in comparison with IL-2-treated patients. Results of the first analysis have been already reported in 2002 after a mean followup of 4 years [8] and updated after a 7-year followup in 2007 [9]. The aim of this work was to update the clinical results after a 14-year followup for the last patient treated and to identify some predictive factors for the clinical response of the so long responder patients.

## 2. Materials and Methods

### 2.1. Trial Design

This study was conducted on the same population as in the former studies published in 2002 and 2007 [8, 9]. Patients aged between 18 and 75 years had to meet the following inclusion criteria: histologically proven primary cutaneous melanoma without any prior systemic adjuvant therapy; clinically apparent regional lymph node recurrence occurring at any time after surgery for primary melanoma regardless of the depth (T1-4N recurrent M0); no sentinel node dissection previously undergone; absence of visceral metastases controlled by physical and radiological exams. Contraception was required in women of childbearing age. Patients were randomized as soon as the histology was confirmed.

Patients were then randomly assigned to receive either two injections of TIL (about 6 and 10 weeks after surgery, according to the duration of the expansion) combined with IL-2 (Proleukin; Chiron) or IL-2 alone. Subcutaneous IL-2 treatment was initiated 6 weeks after lymph node resection in the control arm at the same dose, that is, 6 × 10^6^ IU/m^2^ per day, 5 days a week for 2 weeks. TIL were injected the same day as IL-2 treatment was initiated in the combined arm. One month later, both arms received again the same treatment cycle. After 2 months of adjuvant therapy, patients received no other treatment. Patients were followed up on a regular basis [8].

*Followup*. In both treatment groups, clinical examination, full blood counts, and biochemical analyses were repeated every 15 days during the first 2 months, then every 2 months for 18 months, and finally every 3 months. Liver echography and brain-chest-liver CT scan were performed every 6 months.

The date and site of first recurrence as well as the date and cause of death were recorded. Adverse events were noted and the WHO toxicity scale was used to grade their severity. The study was a monocentric trial to ensure the reproducibility of lymph node excision. This study was approved by the ethical committee of Nantes (Pays de La Loire).

### 2.2. TIL Production by the Invaded LN

In all patients, only one of the metastatic nodes was used for TIL expansion. TIL were minimally cultured for a short period with a low dose of recombinant interleukin-2 (rIL-2) according to a procedure previously described in [10, 11]. Briefly, short-term cultured TIL were isolated by culturing stage III metastatic LN fragments in 12-well tissue culture plates with X-VIVO 15 serum-free medium (BioWhittaker, Walkersville, MD, USA) containing low doses of rIL-2 (150 U/mL) (Eurocetus, Rueil Malmaison, France) and glutamine (1 mM, BioWhittaker) for 10–14 days. *Ex vivo* expanded TIL were derived as follows: 1.8 × 10^6^ short-term cultured TIL were plated at 300 viable lymphocytes/well with irradiated feeder cells (allogeneic peripheral blood leukocytes (PBL) and B-EBV cells: Epstein-Barr-virus-infected B cells) in U-bottom microplates in medium containing low doses of rIL-2 (150 *μ*L). PHA-L (phytohemagglutinin-L or leucoagglutinin) (Difco, Detroit, ML, USA) was added on day 0 (1 *μ*g/mL). Ten days later, lymphocytes were recovered from the culture plates, adjusted to 1 × 10^6^ cells/mL in rIL-2 medium, and transferred in culture cell bags for 10 additional days. The final TIL harvest was obtained by centrifuging, washing, and suspending the TIL in 4% human serum albumin (LFB, Les Ulis, France). A second TIL expansion was performed within one month after the first expansion, using cryopreserved short-term cultured TIL. Aliquots of TIL suspensions injected to patients were cryopreserved to subsequently study their tumor specificity, once the autologous tumor cell line had been established in culture.

The 88 TIL expansions (two expansions for each of the 44 patients) were all performed successfully and then injected to patients. No technical problems were noted during these expansions and all the bacteriological controls were negative. The polyclonal TIL lines were obtained from a tumor invaded lymph node of each patient randomized to receive TIL treatment combined with IL-2, regardless of the tumor specificity of infused populations, which was analyzed retrospectively six/twelve weeks after patient's enrollment, a time required to obtain an autologous melanoma cell line.

### 2.3. Translational Research

In the arm of the 44 patients treated with TILs, the biological material was available for translational research for 31 patients. We obtained melanoma cell lines for 18/31 patients. After lymph node resection, the invaded LN were fragmented to be used to produce therapeutic autologous TIL, for immunohistochemical analysis, to establish the autologous tumor cell line and for pathological examination and BRAF and NRAS mutation analysis. Twelve patients had only one invaded node and 19 patients had more than one invaded node. The primary melanomas of these patients were all of the superficial spreading subtype (SSM) and of stage IIb or IIc corresponding to TNM classification T3b or T4a or T4b/N0/M0 (AJCC 2010).

#### 2.3.1. Characteristics of Injected TILs

*(1) Antibodies and Flow Cytometric Analysis*. The following antibodies were used: PC5 anti-CD3 (clone UCHT1), PE anti-CD8 (clone B9.11), and APC anti-CD4 (clone 13B8.2), all from Beckman Coulter, Marseille, France. Lymphocytes were gated according to their forward and size scatter characteristics, and FACScan analysis was performed using the BD FACS Diva software (BD Biosciences, San Jose, CA, USA).

*(2) Establishment of Melanoma Cell Lines (Obtained for 18/31 Patients)*. Melanoma cell lines were established as previously described in [12, 13] and were successfully established for 18 tumor samples. Briefly, fresh LNs with metastasis were minced into small tumor pieces and plated in a 24-well plate with 1.5 mL RPMI per well (Roswell Park Memorial Institute) supplemented with 10% fetal calf serum (FCS). Plates were placed at 37°C in a humidified incubator with 5% CO_2_ and observed under a light microscope every week and subcultured when necessary.

*(3) Cytokine Production Assay for Assessing the Proportion of Tumor-Specific TIL*. The proportion of tumor-reactive TIL was determined through the measurement of the proportion of interferon-gamma- (IFN-*γ*-) secreting T cells within the TIL stimulated with the autologous melanoma cell line, as described previously in [11]. Briefly, lymphocytes were stimulated with autologous melanoma cells in the presence of brefeldin A for 6 h at 37°C in 5% CO_2_ humidified atmosphere. Fixed stimulated lymphocytes were stained for IFN-*γ* production using the method described by Jung et al. [14]. After staining, cells were resuspended in PBS and 10^5^ events were analyzed on a FACSCalibur flow cytometer using the BD FACS Cell Quest Pro software (BD Biosciences, San Jose, CA, USA). T-cell responses were considered significant when the mean fluorescence labeling of TIL stimulated with the autologous tumor cell line exceeded, by at least half a log, the mean fluorescence of the background responses of nonstimulated TIL and/or of TIL stimulated with a HLA-mismatched melanoma line. A value of 0.3% was considered as the significance threshold.

#### 2.3.2. Melanoma Tissue Analysis

*(1) Immunohistochemistry on Tumor LN*. Immunohistochemistry (IHC) was performed using the streptavidin/peroxidase technique as previously described in [15]. Deep-frozen sections were incubated for 30 minutes at room temperature with the primary antibody. Three different monoclonal antibodies were used to explore the expression of T-cell-associated molecules: anti-Foxp3 (clone 236A/E7, 5 *μ*g/mL, eBioscience, San Diego, CA, USA), anti-PD-1 (clone J105, 5 *μ*g/mL, eBioscience), and anti-PD-L1 (clone MIH1, 5 *μ*g/mL, eBioscience). Negative controls were done using a mouse monoclonal immunoglobulin-G1 (IgG1) isotype control (DakoCytomation). Slides were read with a Leica microscope (magnification ×25). In each immunostained serial section, the entire tumour area was assessed. Each score was evaluated using a five-point scale: absence of expression, weak (1–25% of positive cells), moderate (26–50%), intermediate (51–75%), and strong expressions (>75%) were represented, respectively by levels 0, 1, 2, 3, and 4. Photographs were taken using a digital SLR Nikon D70S camera. To avoid the subjectivity of the reading, all the slides were blindly read by two independent examiners.

*(2) DNA Extraction*. Serial sections were cut from each paraffin block and placed on glass slides. The first 3 **μ**m thick section was stained with hematoxylin and eosin (H&E) for histopathological examination. The 2–5 following 10 **μ**m thick sections were processed for DNA extraction. To enrich the analyzed specimen with tumor cells, tumor areas highlighted by a pathologist on H&E preparation were macrodissected using single-use sterilized scalpels. DNA was extracted after paraffin removal and macrodissection using the Forensic kit and an iPrep system according to the manufacturer's recommendations (Invitrogen, Life Technologies SAS, Villebon sur Yvette, France). DNA concentration was quantified by spectrophotometry (NanoDrop ND-100 instrument, Thermo Fisher Scientific, Waltham, MA, USA) and normalized to 5 ng/*μ*L.

*(3) Detection of BRAF V600 and NRAS Q61 Mutations*. The most frequent BRAF mutations were detected using an allele-specific amplification as previously described in [16] with minor modifications. Two forward primers with variations in their 3′ nucleotides to be specific either of the wild-type (V600; AGG TGA TTT TGG TCT AGC TAC AGT) or the mutated variant (600E; AGG TGA TTT TGG TCT AGC TAC AGA) were designed and used in separate reactions with a common reverse primer (AS; ATG GAT CCA GAC AAC TGT TCA AAC). Each sample was also analyzed by conventional Sanger DNA sequencing using BRAF 15F (5′-TCA TAA TGC TTG CTC TGA TAG GA-3′) and 15R (5′-GGC CAA AAA TTT AAT CAG TGG A-3′) primers for both amplification and sequencing. NRAS exon 2 mutations were analyzed by conventional Sanger DNA sequencing using NRAS 2F (5′-CCC CCA GGA TTC TTA CAG AA-3′) and 2R (5′-ATA CAC AGA GGA AGC CTT CG-3′) primers for both amplification and sequencing.

These assays allowed detecting BRAF V600 or NRAS Q61 alterations when they were present in at least 10% of cells.

### 2.4. Statistical Analysis

The primary and the secondary endpoints were, respectively, the duration of the disease-free survival and overall survival.

The Kaplan-Meier estimates and log-rank tests were used for the main analysis of the effectiveness. The log-likelihood ratio test and Wilcoxon rank tests were used to assess different factors in translational research. The Cox model was used to adjust treatment comparison on baseline characteristics known to have a prognostic significance, namely, Breslow thickness (<1.5 mm; >1.5 mm), capsular breaking, number of detectable regional nodes (1, >1), sex, and age (<50 yr, 50 yr). Assumption of proportional hazards was checked for all factors. Interactions between the treatment and the number of detectable regional nodes were tested.

## 3. Results

### 3.1. Clinical Long-Term Analysis

The following analysis was based on data collected in May 2013, when the first and the last patients treated had completed, respectively, 19.2 and 14.4 years of followup.

*Study Population*. Eighty-eight patients were enrolled in the study, with 44 subjects in each group; all patients received one of the two treatments and were assessed [8, 9]. The median followup was of 16.7 years. Patient main characteristics are summarized in Table 1.

### 3.2. Safety

The main adverse events experienced by patients were transient and reversible (e.g., fever, asthenia, flu-like symptoms, headache, nausea, and vomiting) in few hours for TIL and few days for IL-2. No grade 3/4 toxicity or drug-related mortality was observed. No patient withdrawal from the study was due to adverse events. No cardiovascular symptoms, haematological toxicity of grade 3 or 4, or increase in liver enzyme levels of grade 3 or 4 was noted [8, 9].

No alive patients treated with TIL experienced an autoimmune disease or induction of EBV after this long followup although TIL were obtained with LAZ cell line as feeder.

### 3.3. Relapse-Free Survival (RFS)

**Figure 1:**
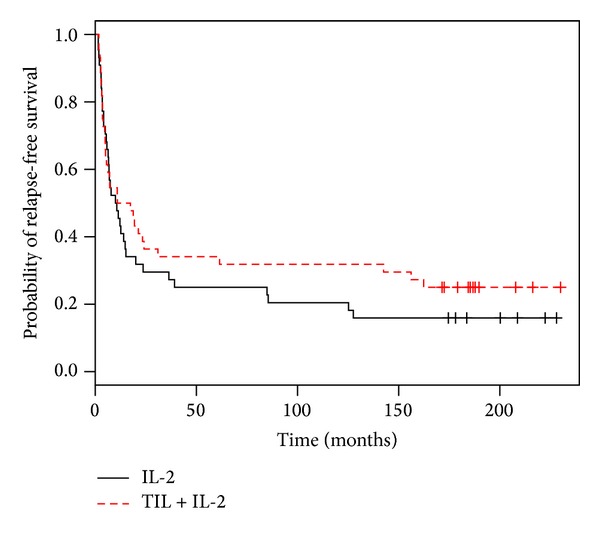
Relapse-free survival in the TIL + IL-2 group: this difference was not significant (*P* = 0.45; log-rank test); 16.7 years of median followup.

Thirty-three recurrences were recorded in the TIL + IL-2 group and 37 in the control group (Table 2). The median relapse-free survival was of 14.2 months for the TIL group and 10.3 months for the control group. As for the results obtained in 2007, the crude difference was not significant (log-rank test; *P* = 0.35; Figure 1). However, the Cox model adjusted on the others factors, including the significant interaction between the treatment and the number of invaded lymph nodes, showed for the first time a global and independent significant effect of the treatment (*P* = 0.008) (Table 3(a)).

**Figure 2:**
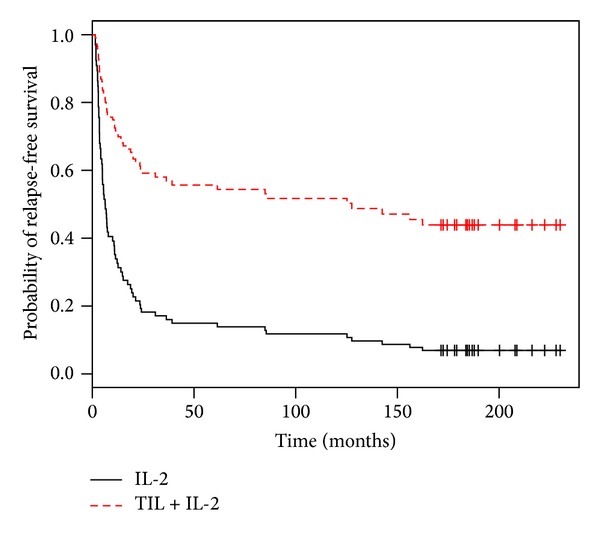
Cox model curves when the Breslow is >1.5, a capsular breaking is present, and only one lymph node is invaded.

Figure 2 represents Cox survival curves when only one node was invaded, the Breslow thickness was ≥1.5, and a capsular breaking was present.

### 3.4. Overall Survival (OS)

**Figure 3:**
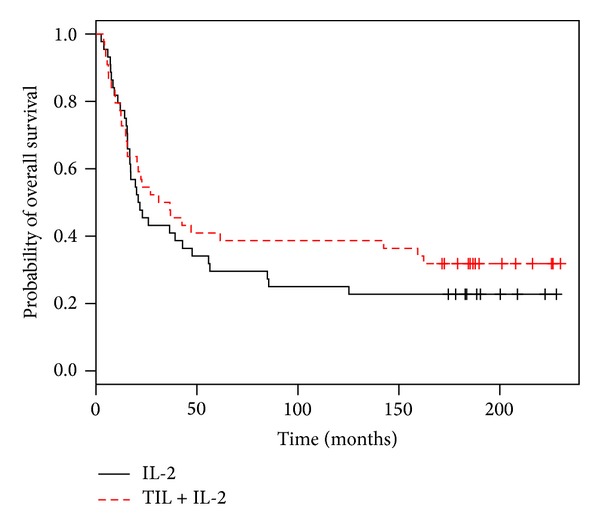
The overall survival rate between the TIL + IL-2 group and IL-2 group was not significantly different (*P* = 0.39; log-rank test); 16.7 years of median followup.

Thirty out of the 44 patients died in the TIL + IL-2 group, and 34 out of the 44 in the control group. The median survival was of 33.4 months in the TIL + IL-2 group and 30.6 months in the IL-2 control group. This difference was not significant (*P* = 0.39, log-rank test; Figure 3).

**Figure 4:**
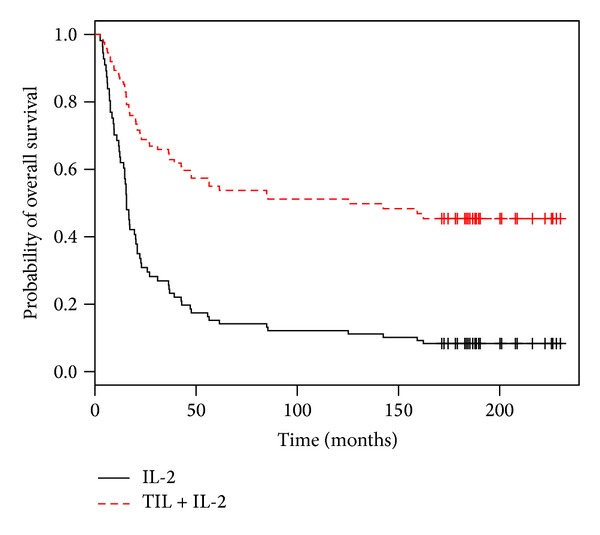
Cox survival curves when only one node is invaded, the Breslow is ≥1.5, and a capsular breaking is present.

The Cox model adjusted on the others factors was comparable with the relapse-free survival model. There was an association between the treatment and the number of tumor-invaded nodes (*P* = 0.006). For the first time, this analysis showed a global and independent significant effect of the treatment (*P* = 0.015) (Table 3(b)).  Figure 4 represents Cox survival curves when only one node was invaded, the Breslow thickness was ≥1.5, and a capsular breaking was present.

### 3.5. TIL Obtained from the Invaded LN

In the arm of the 44 patients treated with TILs, the biological material was available for translational research for 31 patients. Melanoma cell lines were obtained for 18/31 patients. The 31 samples of melanoma-invaded LN were used for producing therapeutic autologous TIL. There was no significant difference in the proportion of total TIL infused to patients between patients with only one invaded LN and those with more than one invaded LN (1st expansion, Wilcoxon, *P* = 0.8392; 2nd expansion, Wilcoxon, *P* = 0.5565) (Table 4). Moreover, the proportion of TIL infused to patients was not associated with disease recurrence (1st expansion, RFS, *P* = 0.526, OS, *P* = 0.682; 2nd expansion, RFS, *P* = 0.888, OS, *P* = 0.926). In the group of patients with more than one invaded LN, only one of the metastatic nodes was used for TIL expansion.

### 3.6. Phenotype of Infused TIL

There was no significant difference in the phenotype of expanded TIL infused to patients between the two groups, regarding CD3/CD4 and CD3/CD8 double positive cells (Table 4) (CD3/CD4 positive cells, 1st expansion Wilcoxon, *P* = 0.6263; 2nd expansion, Wilcoxon, *P* = 0.9515; CD3/CD8 positive cells, 1st expansion, Wilcoxon, *P* = 0.792; 2nd expansion, Wilcoxon, *P* = 1). The CD4/CD8 ratio was not different between the two groups (1st expansion, Wilcoxon, *P* = 0.6555; 2nd expansion, Wilcoxon, *P* = 0.9523). Moreover, the phenotype of TIL infused to patients was not associated with disease recurrence (CD3/CD4 positive cells, 1st expansion, RFS, *P* = 0.815, OS, *P* = 0.551; 2nd expansion, RFS, *P* = 0.285, OS, *P* = 0.787; CD3/CD8 positive cells, 1st expansion, RFS, *P* = 0.32, OS, *P* = 0.927; 2nd expansion, RFS, *P* = 0.225, OS, *P* = 0.703; CD4/CD8 ratio, 1st expansion, RFS, *P* = 0.237, OS, *P* = 0.549; 2nd expansion, RFS, *P* = 0.252, OS, *P* = 0.41).

### 3.7. Tumor-Reactive TIL

The same 31 samples of melanoma-invaded LN, from which TIL were obtained, were used to assess the proportion of T cells reactive to the autologous melanoma cell line within the TIL infused to patients (Table 4). Both RFS and OS were associated with the presence of tumor-reactive T cells: the duration of survival without relapse or death was increased by the injection of melanoma-reactive TIL (*P* = 0.019; *P* = 0.00468). In a monovariate analysis, the Kaplan-Meier statistical analysis showed that both RFS and OS were significantly longer for patients receiving tumor-reactive TIL. There was no significant difference in the % of reactive T cells infused to patients between patients with only one invaded LN and those with more than one invaded LN (1st expansion, Wilcoxon, *P* = 0.2322; 2nd expansion, Wilcoxon, *P* = 0.2146). Moreover, the proportion of reactive T cells infused to patients was not associated with disease recurrence (1st expansion, RFS, *P* = 0.739, OS, *P* = 748; 2nd expansion, RFS, *P* = 0.192, OS, *P* = 0.572).

### 3.8. Immunohistochemistry on Tumor LN

A total of 31 tissue specimens of tumor-invaded LNs from stage IIIb (AJCC) melanoma patients were available for assessing the protein expression using immunohistochemistry.

### 3.9. Foxp3

We observed that the expression of the nuclear transcription factor forkhead/winged helix transcription factor 3 (Foxp3) was significantly increased in LN samples from stage III patients with more than one invaded LN compared to patients with only one invaded LN (mean expression: 0.947 and 0.167, resp.; Wilcoxon, *P* = 0.0003) (Table 5).

Moreover, the expression of Foxp3 was associated with disease recurrence: patients with tumors expressing Foxp3 had a shorter response duration to adoptive TIL transfer. Foxp3 was thus associated with a shorter RFS (*P* = 0.01) and OS (*P* = 0.045).

### 3.10. PD-1 and PD-L1

The expression of PD-1 was significantly increased in LN samples from stage III patients with more than one invaded LN compared to patients with only one invaded LN (5/8 and 1/9 samples expressed PD-1, resp.; Wilcoxon, *P* = 0.049). Moreover, the expression of PD-1 was not associated with disease recurrence (RFS, *P* = 0.49; OS, *P* = 0.46) and no significant difference was observed between the two groups (Table 5) (RFS, *P* = 0.48; OS, *P* = 0.66).

### 3.11. BRAF and NRAS Mutation Status

The presence of both BRAF codon V600 and NRAS exon 2 mutations was not significantly different between patients with more than one invaded LN compared to patients with only one invaded LN (see Supplementary File S1 available online at http://dx.doi.org/10.1155/2014/186212) (BRAF codon V600 mutation, *P* = 0.38; NRAS exon 2 mutation, *P* = 0.16). Moreover, the presence of BRAF and NRAS mutations was not associated with disease recurrence (BRAF mutation, RFS, *P* = 0.78, OS, *P* = 0.59; NRAS mutation, RFS, *P* = 0.11, OS, *P* = 0.07). However, it should be noted that due to the age of the FFPE tissue samples (stored between 1994 and 1998), the analyses were not contributive for 8 tissue samples for the BRAF mutation and for 12 tissue samples for the NRAS mutation.

## 4. Discussion

The present paper includes the last analysis of our study conducted in a cohort of patients treated either with IL-2 or adoptive T-cell therapy (TILs + IL-2) as adjuvant treatment in stage III melanoma patients. The median followup of 16.7 years is the longest clinical followup reported so far in the field of adoptive T-cell therapy (14.3–19.2).

Two previous analyses were published in 2002 and 2007 [8, 9] showing a clinical benefit of TILs in the subgroup of patients with only one invaded lymph node.

This new 3rd analysis confirms the association between the treatment and the number of invaded lymph nodes. The new major finding of this last analysis, compared to the two previous ones, is the evidence of the beneficial effect of TIL treatment without using a subgroup analysis. After a mean followup of 16.6 years, the median survival remained improved in the TIL + IL-2 group, with 33.8 months compared to 21.3 months in the IL-2 group. However, given the small number of patients with a single invaded lymph node, these results need to be confirmed in a larger cohort. For this purpose, a phase III clinical trial including only patients with one invaded lymph node and treated with TIL is ongoing in our unit.

Regarding the safety, this long followup confirmed the safety of TILs infusions. In particular, no visceral or cutaneous autoimmune illness such as thyroiditis or uveitis or vitiligo was noted. Given that the TAA expressed by tumor cells are also expressed by normal healthy cells (such as retinal cells expressing the Melan-A antigen) and that the specific T cells can also destroy normal cells [17], we focused the monitoring of adverse events on autoimmune reactions during this very long clinical followup of patients treated with TIL.

Furthermore, we confirmed that the use of an irradiated EBV cell line for stimulating T lymphocytes during expansion did not induce any EBV infection or reactivation.

These clinical findings were completed by a translational research performed in 31/44 patients receiving TILs for whom both infused TIL and tissue samples, from which TILs were obtained, were available. The objective was to identify predictive markers for the clinical response using these two types of material taking advantage of a very long patient followup (15 years) allowing to consider that patients not relapsing were cured.

The first part of the study was performed on TIL expansion and confirmed that the presence of reactive T lymphocytes within the expansion was a strong predictive factor for treatment benefit. We confirmed that both the RFS and OS of patients treated with TIL correlated with the presence of tumor-reactive T cells within the TILs infused to patients. The importance of specificity of the transferred TILs had already been demonstrated in our two previous articles showing that the infusion of Melan-A/MART-1 or Meloe-1 reactive TIL was associated with a longer relapse-free survival for HLAA2 patients presenting a low tumor burden [18, 19].

However, the proportion of injected TIL was not significantly different between patients with only one invaded LN and those with more than one invaded LN. Moreover, the proportion of TIL infused to patients was not associated with disease recurrence. We thus confirmed our results which were previously reported in [11, 20, 21].

Regarding the melanoma samples, Foxp3 expression was confirmed as a predictive marker for survival in patients treated with TILs. Foxp3 remains the most specific marker of Tregs to date. Our group already quantified Foxp3 expression using qPCR and Foxp3 appeared as an independent prognostic factor for PFS in stage III melanoma patients with invaded lymph nodes [22]. We also reported that Foxp3 expression is increased in LN tissue samples from patients with more than one invaded LN, compared to patients with only one invaded LN [21]. Similarly, Hamid et al. reported significant associations between the clinical activity and the high baseline expression of two immune-related genes, Foxp3 and indoleamine 2,3-dioxygenase (IDO), in advanced melanoma patients treated with anti-CTLA-4 Ipilimumab [23]. In a pilot study conducted in 15 patients treated with anti-CTLA-4 Tremelimumab, the clinical response was also associated with an increase in tumor infiltration by CD8+ TIL and a variable association with CD4+ TIL [24]. In this study, the presence of Foxp3+ cells was variably associated with a positive response. While analyzing the safety of surgery and the immune phenotype of the resected tumors under Ipilimumab, Gyorki et al. reported an increase in CD4+Foxp3+ T-regulatory cell proportion and a 2.8-fold lower CD8+/CD4+Foxp3+ ratio in the tumor compared with the blood, in progressive tumors, suggesting a possible mechanism of immune escape [25]. Thus, Foxp3 appears overall as a predictive marker for immunological approaches in melanoma.

Regarding the expression of the negative costimulatory molecules such as PD-1 and PD-L1, we observed a higher expression of PD-1 in patients with more than one invaded lymph node arguing that in melanoma advanced stage an increase in tumor burden was associated with an induced expression of the coinhibitory molecule PD-1 in the T lymphocytes which could explain the loss of efficacy of TILs.

Moreover, the presence of BRAF and NRAS mutations was not different between the two groups of patients. Our data also did not show any effect of the BRAF or NRAS mutational status neither on the RFS nor on the OS.

These results which were obtained after a more than 15-year clinical followup show clearly the interest of using adoptive TIL transfer in the adjuvant setting for melanoma patients. However, even at this very early stage of the disease, the clinical benefit was only experienced by patients with low tumor burden and thus having still a powerful immune system with a low or absent immunotolerance state of the tumor microenvironment notably induced by an increase in Tregs and the expression of PD-1 in infiltrating T lymphocytes.

In the advanced stage of melanoma, the adoptive TIL transfer needs to be potentiated to be more effective.

Thus combined immune-stimulatory strategies appear to be of particular interest. Antibodies targeting PD-1 and/or CTLA-4 (blocking the negative costimulatory molecules and then boosting the T-cell function) could improve the adoptive transfer efficiency and lead to a clinical benefit in patients with high tumor burden. Similarly, the combination of TIL with BRAF inhibitors (inducing cell apoptosis and the release of antigens which initiate T-cell activation) or BRAF/MEK inhibitors could be of interest in the treatment of metastatic melanoma.

## Supplementary Material

Schematic representation of the protocol used for the different starvation conditions tested.

## Figures and Tables

**Table 1:** Patient characteristics according to the treatment group.

	TIL + IL-2 (*n* = 44)	IL-2 (*n* = 44)
Primary tumor site*		
Head	7	0
Trunk	20	22
Limbs	16	20
Extremities	1	1
Breslow**		
<1.5	9 (0.8 ± 0.1)	9 (1.2 ± 0.1)
>1.5	31 (4.3 ± 0.5)	33 (3.6 ± 0.3)
Invaded nodes		
=1	15	19
>1	29	25
Capsular breaking***		
Yes	23	22
No	20	21

*Primary tumor site unknown for one IL-2 patient, **data not available for 4 TIL patients and 2 IL-2 patients, and ***data not available for 1 TIL patient and 1 IL-2 patient.

**Table 2:** Proportion of relapse according to the treatment and number of invaded nodes (1 or >1). Data obtained in June 2000, April 2006, and March 2013.

	TIL + IL-2			IL-2			All		
	2000	2006	2013	2000	2006	2013	2000	2006	2013
1 invaded node	5/15		7/15	13/19		16/19	18/34		23/34
	(33.3%)		(46.66%)	(68.42%)		(84.21%)	(52.94%)		(67.64%)
>1 invaded node	24/29		26/29	18/25	19/25	21/25	42/54	43/54	47/54
	(82.75%)		(89.65%)	(72%)	(76%)	(84%)	(77.8%)	(79.6%)	(87.03%)

All	29/44		33/44	31/44	32/44	37/44	60/88	61/88	70/88
	(65.9%)		(75%)	(70.5%)	(72.7%)	(84.09%)	(68.18%)	(69.3%)	(79.54%)

**Table:** (a)

	IC 95% lower	OR	IC 95% upper	*P* value
TIL + IL-2	0.121	0.297	0.731	0.0082
Lymph nodes > 1	0.506	0.971	1.865	0.930
Breslow > 1.5	1.242	2.477	4.943	0.0100
Capsular effraction	0.973	1.571	2.535	0.0654
TIL + IL-2 ∗ lymph nodes > 1	1.732	5.087	14.945	0.0031

The Cox model showed a significant treatment effect (*P* = 0.0082) and confirmed the significant interaction between TIL + IL-2 and the number of invaded lymph nodes.

**Table:** (b)

	IC 95% lower	OR	IC 95% upper	*P* value
TIL + IL2	0.117	0.305	0.794	0.0151
Lymph nodes > 1	0.419	0.8281	1.634	0.586
Breslow > 1.5	1.010	2.025	4.067	0.0471
Capsular effraction	1.239	2.085	3.508	0.0056
TIL + IL-2 ∗ lymph nodes > 1	1.604	5.019	15.704	0.0057

The Cox model for the overall survival was comparable with that of the relapse-free survival.

**Table 4:** Proportion, phenotype, and fractions of tumor-reactive lymphocytes of large-scale expanded TIL from melanoma-invaded lymph nodes from 31 patients.

Patients	Relapse^d^	Number of infused TIL × 10^9^		% CD3/CD4-positive^b^		% CD3/CD8-positive^b^		Ratio of CD4/CD8		% IFN-*γ*-positive lymphocytes^c^	
		E1^a^	E2	E1	E2	E1	E2	E1	E2	E1^a^	E2
M88*	+	6	0.7	27	39	71	59	0.4	0.7	ND	0.4
M98*	+	33.5	21.4	85	93	14	7	6.1	13.3	NA	NA
M99	−	1.7	0.8	60	33	38	61	1.6	0.5	NA	NA
M100	+	11.6	6	94	95	5	4	18.8	23.8	NA	NA
M103*	+	7.65	27.6	43	57	66	58	0.7	1.0	NA	NA
M110	−	1.13	1.05	78	84	18	18	4.3	4.7	0.0	0.0
M113	−	10	11.2	36	75	75	28	0.5	2.7	10.3	0.8
M117	+	1.4	32	30	3	70	97	0.4	0.03	ND	2.50
M125	−	12	13.2	71	65	30	36	2.4	1.8	0.0	2.4
M126	−	10.2	10.8	55	39	40	56	1.4	0.7	NA	NA
M132	−	8.3	12.2	39	20	58	80	0.7	0.3	0.0	0.0
M134	+	13.5	9	55	62	45	38	1.2	1.6	0.7	0.0
M139*	−	12.3	13.4	53	60	42	34	1.3	1.8	NA	NA
M148	−	15.2	3.1	39	46	57	55	0.7	0.8	NA	NA
M158	−	8	10.2	30	31	70	63	0.4	0.5	0.0	0.0
M170*	+	9	0.53	30	32	67	67	0.4	0.5	1.2	ND
M171	−	10	12	48	42	52	58	0.9	0.7	0.0	0.0
M174*	+	9.5	4.7	80	70	20	30	4.0	2.3	NA	NA
M180*	+	5.5	3.5	29	19	68	80	0.4	0.2	NA	NA
M182	+	0.576	3.1	87	25	13	71	6.7	0.4	0.0	0.7
M187	+	8	10	16	24	86	76	0.2	0.3	2.9	0.4
M192	−	10	15.6	49	82	51	18	1.0	4.6	NA	NA
M193	−	12	5.2	14	27	86	73	0.2	0.4	3.5	4.0
M196*	−	8.6	14	40	22	58	74	0.7	0.3	1.0	0.0
M197*	+	14	1	48	32	52	68	0.9	0.5	2.9	0.0
M199	−	5	3.3	25	14	75	86	0.3	0.2	2.7	2.4
M204	−	9.5	16.5	92	88	8	17	11.5	5.2	0	3.4
M209*	−	6.8	6.2	63	71	36	29	1.8	2.4	NA	NA
M212	−	7.8	11	52	33	47	57	1.1	0.6	0.4	1.0
M213*	+	9.1	8.5	24	24	76	72	0.3	0.3	NA	NA
M215*	+	8.8	5.5	45	68	46	31	1.0	2.2	NA	NA

*Melanoma patients bearing only one metastatic lymph node.

^a^E1 and E2 were TIL populations obtained and reinjected to the patient from, respectively, the first and the second *ex  vivo* expansions.

^b^Percentages of CD-positive TIL were estimated by membrane labeling. Cells were analyzed on a FACScan.

^c^Percentages of IFN-*γ* secreting TIL were estimated by intracellular labeling. TIL were stimulated 6 h by autologous melanoma cells in presence of brefeldin A.

Then, cells were fixed, permeabilized, stained for cytokine production, and analyzed on a FACSCalibur.

^d^Relapse of patients ((−): patients who relapsed; (+): patients who did not relapse).

**Table 5:** Relationship between some predictive factors and the relapse-free survival, overall survival, and tumor burden.

	Relapse-free survival	Overall survival	1 LN versus >1 LN
	*P*	*P*	*P*
Im FOXP3	0.01	0.045	0.0003
Im PD-1	0.49	0.46	0.049
Im PD-L1	0.48	0.66	0.57
BRAF	0.78	0.59	0.38
NRAS	0.11	0.07	0.16

## Abbreviations

IL-2: Interleukin-2
rIL-2: Recombinant interleukin-2
RFS: Relapse-free survival
OS: Overall survival
FDA: Food and Drug Administration
CTLA-4: Cytotoxic T-Lymphocyte Antigen 4
TIL: Tumor infiltrating lymphocytes
TAA: Tumor associated antigen
IFN-*γ*: Interferon-*γ*
PD-1: Programmed-death-1
PD-L1: Programmed-death ligand 1
Foxp3: Forkhead box transcription factor 3
TGF-*β*: Transforming growth factor-*β*
IL-10: Interleukin-10
AJCC: American Joint Committee on Cancer
PHA-L: Phytohemagglutinin L
EBV: Epstein Barr virus
LN: Lymph node
SSM: Superficial spreading melanoma
TNM: Tumor lymph Nodes Metastasis staging system
RPMI: Roswell Park Memorial Institute medium
FCS: Fetal calf serum
FACS: Fluorescence activated cell sorter
AE: Adverse event
CD: Cluster of differentiation
IDO: Indoleamine 2,3-dioxygenase
MHC: Major histocompatibility complex.
