# Supplementary material for: Adoptive TIL Transfer in the Adjuvant Setting for Melanoma: Long-Term Patient Survival
Source: J Immunol Res. 2014 Jan 8;2014:186212. doi: 10.1155/2014/186212 (PMC3987883; doi:10.1155/2014/186212)
Supplement: Supplementary file 1 — Schematic representation of the protocol used for the different starvation conditions tested. [file 186212.f1.ppt]

## Slide 1
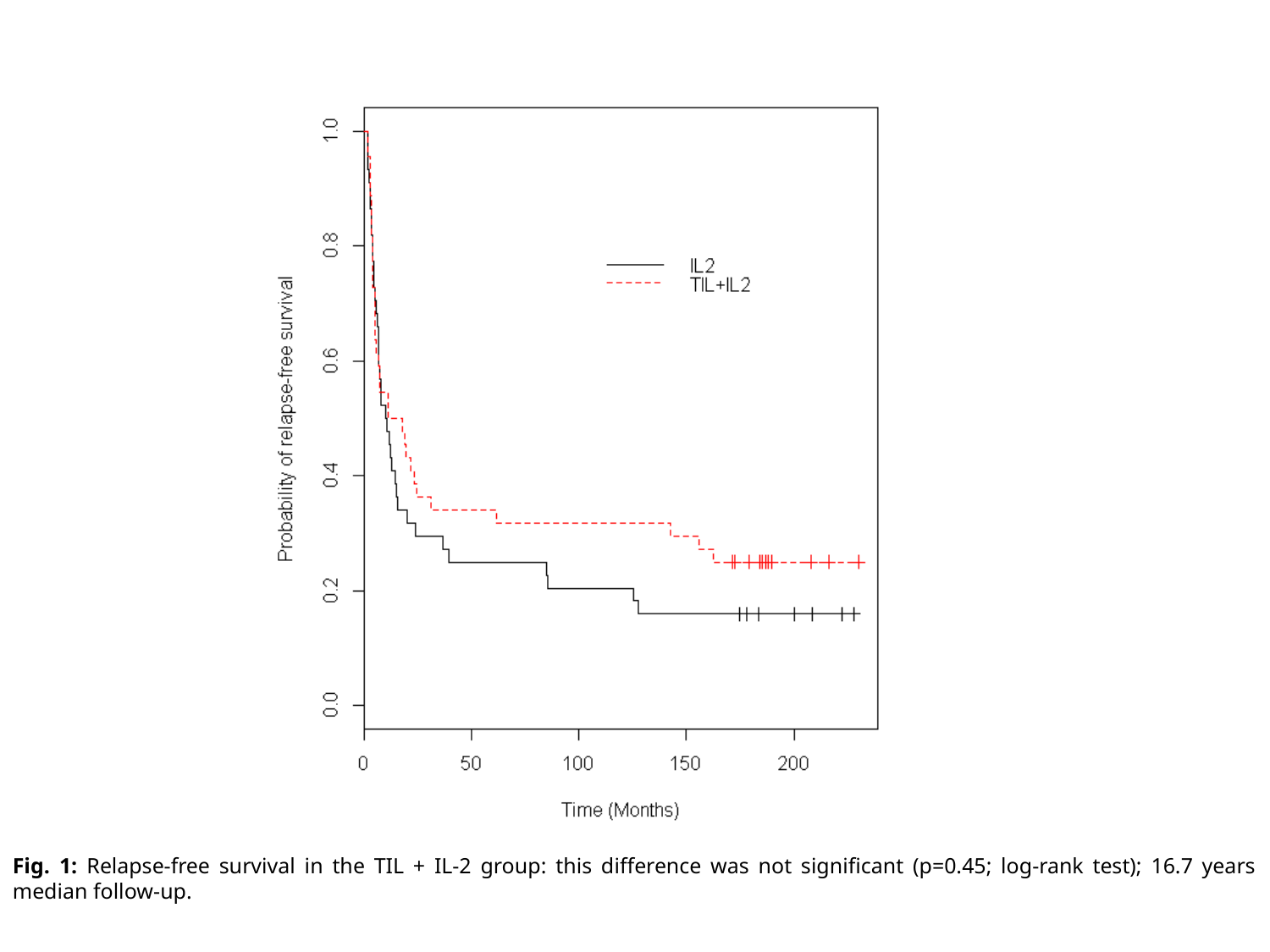

Fig. 1: Relapse-free survival in the TIL + IL-2 group: this difference was not significant (p=0.45; log-rank test); 16.7 years median follow-up.

## Slide 2
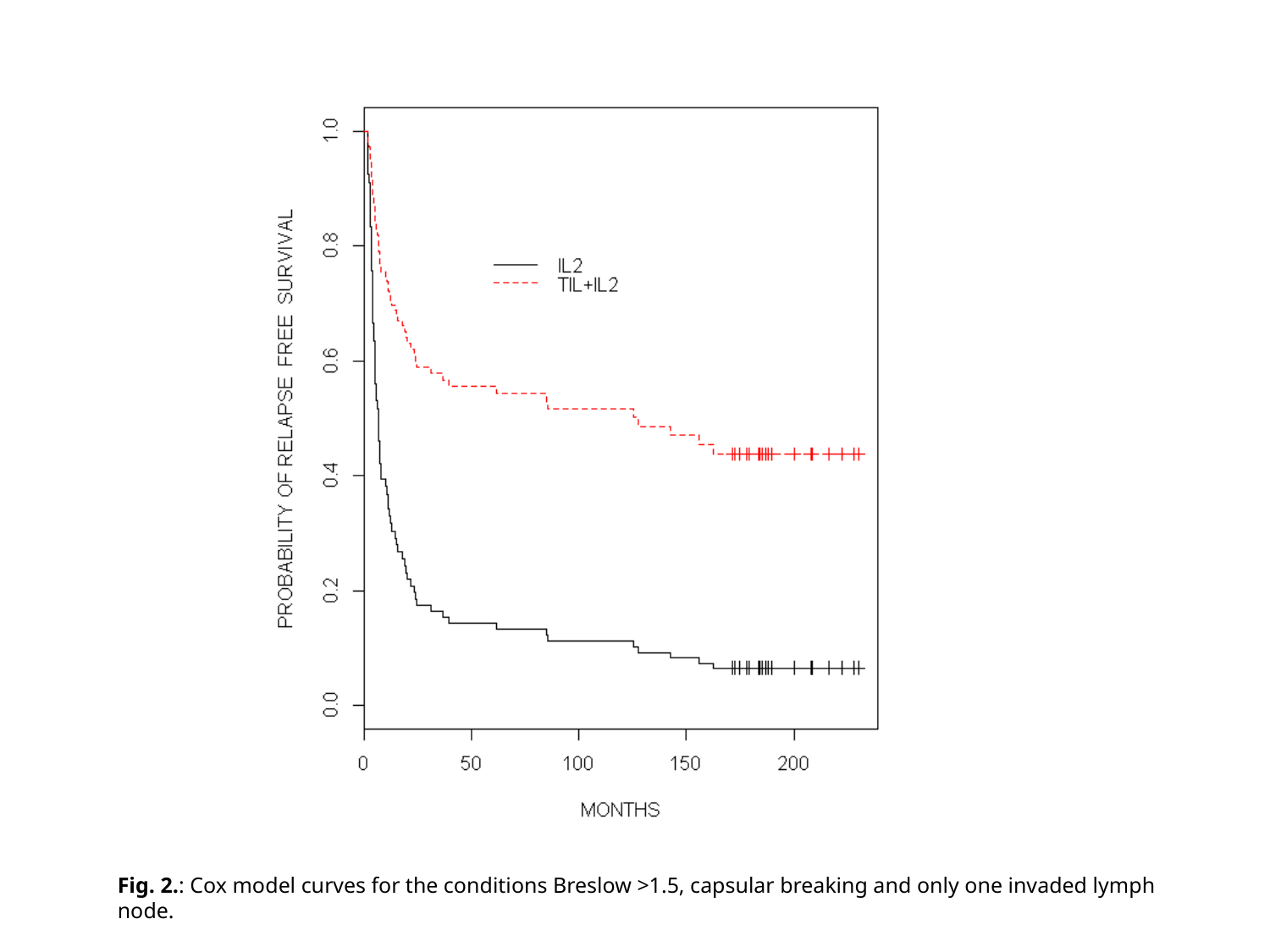

Fig. 2.: Cox model curves for the conditions Breslow >1.5, capsular breaking and only one invaded lymph node.

## Slide 3
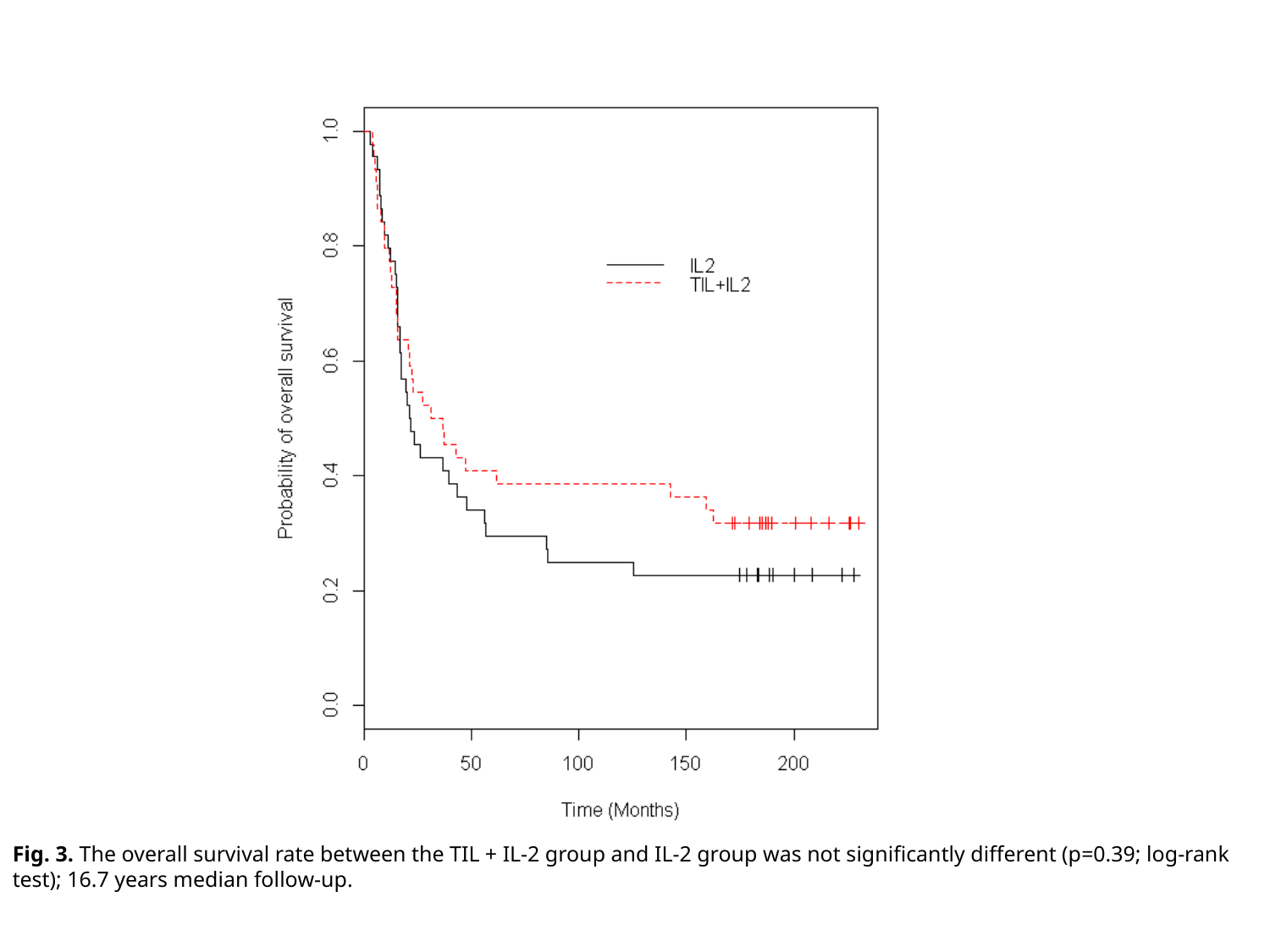

Fig. 3. The overall survival rate between the TIL + IL-2 group and IL-2 group was not significantly different (p=0.39; log-rank test); 16.7 years median follow-up.

## Slide 4
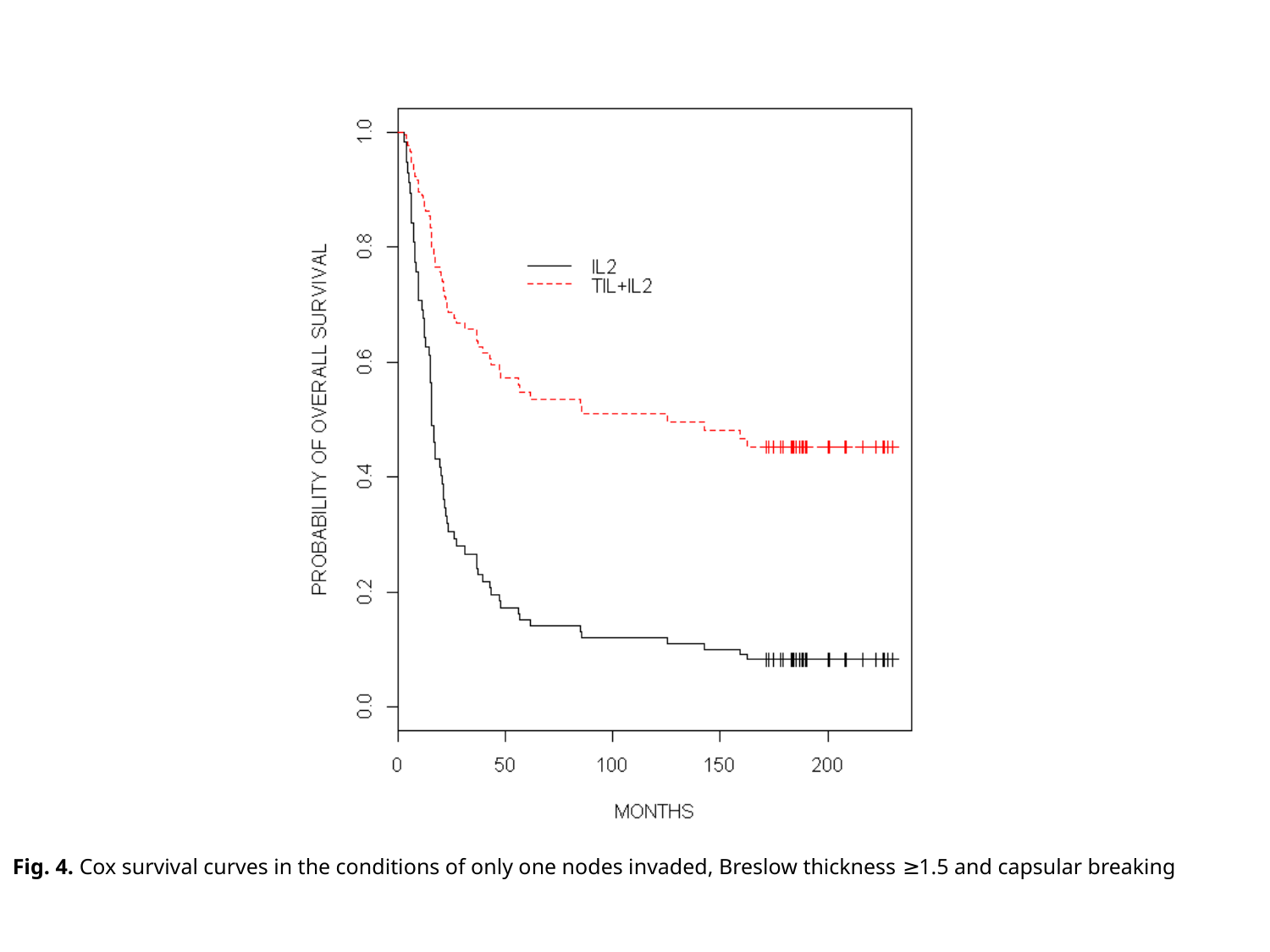

Fig. 4. Cox survival curves in the conditions of only one nodes invaded, Breslow thickness ≥1.5 and capsular breaking
